# Ethical challenges and dilemmas in the rationing of health commodities and provision of high-risk clinical services during COVID-19 pandemic in Ethiopia: the experiences of frontline health workers

**DOI:** 10.1186/s13010-023-00136-6

**Published:** 2023-06-28

**Authors:** Tsegaye Melaku, Ahmed Zeynudin, Sultan Suleman

**Affiliations:** 1 School of Pharmacy, Institute of Health, Jimma University, Jimma, Ethiopia; 2 School of Medical Laboratory Sciences, Institute of Health, Jimma University, Jimma, Ethiopia

**Keywords:** Coronavirus disease 2019, Ethical dilemma, Rationing, Ethiopia

## Abstract

**Background:**

Ethical reasoning and sensitivity are always important in public health, but it is especially important in the sensitive and complex area of public health emergency preparedness. Here, we explored the ethical challenges, and dilemmas encountered by frontline health workers amid the coronavirus disease-19 (COVID-19) pandemic in Ethiopia.

**Methods:**

A nationwide survey was conducted amongst the frontline health workers from nineteen public hospitals. Health workers were invited to respond to a self-administered questionnaire. Data were weighted and analyzed using descriptive statistics.

**Results:**

Of the 285 frontline health workers to whom questionnaires were distributed, 217 of them gave their responses (response rate 76.1%). Respondents frequently reported encountering rationing dilemmas on health commodities directly used for the prevention and treatment of COVID-19. Most (83.9%) of the health workers agreed that they encountered ethical challenges very frequently or frequently. Almost all [215(99.1%)] claimed that the limitation of resources was directly used for the treatment and prevention of COVID-19. The frequency of difficulty in the provision of essential clinical services varied between 77% and 98.7% for different services. More than half of the study participants reported that they had encountered difficulty in the provision of clinical care on a daily or weekly basis. Regarding rationing strategies, isolating COVID-19 treatment units and limiting admission were the most frequent rationing strategies used by two-thirds of health workers on a daily or weekly basis.

**Conclusion:**

Front-line health workers encountered numerous ethically challenging situations during COVID-19. More than half of health workers reported that they encountered ethical challenges in rationing the resources and delivery of different clinical services such as family planning services, maternal and childcare, immunization, and chronic care. With limited resources such as ventilators and hospital beds, healthcare providers have been faced with the difficult task of deciding who gets access to these resources and who doesn't. Overall, the COVID-19 pandemic has presented numerous ethical challenges for healthcare providers, highlighting the importance of ethical considerations in healthcare delivery. By being aware of these dilemmas and having policies in place to address them, healthcare providers can ensure that they are providing the best possible care to their patients while upholding ethical standards.

## Introduction

One of the features of public health emergencies is that health needs exceed the available human and material resources [1]. These difficulties are not uncommon in low-resource settings, such as Ethiopia, which has very weak supply chains and limited resources. Difficult decisions need to be taken as to how, when, where, and to whom resources should be allocated. Clinical science offers valuable information to aid these decisions, but science alone is inadequate [2]. The use of ethical frameworks to direct decision-making can help to mitigate some of the unintended and inevitable collateral damage caused by the coronavirus disease-19 (COVID-19) outbreak [3]. The inclusion of ethics in pandemic response plans might make them tools for fostering cooperation and trust at a time when societies will undoubtedly face significant challenges. Using ethical frameworks to guide decisions will increase confidence that the principles described within them, such as accountability, transparency, and trust, will be carefully considered in decision-making [4].

In the context of COVID-19, resource rationing decisions go beyond those specifically connected to patient care. For instance, health officials may need to ration personal protective equipment (PPE) for health workers [5] and hospital administrators must consider how their limited number of health workers should be distributed [6]. Despite their importance in specific decisions, clinical facts and measurements cannot resolve ethical conundrums. Decisions about the distribution of scarce medical equipment, such as a ventilator, to patients for whom its use is clinically advised, must be made in the context of triage. Furthermore, such decisions could not be made ad hoc by individual physicians or organizations because they would be inconsistent and arbitrary [7]. Instead, guidelines are required, and various considerations must be made to make ethical decisions [8].

The urgency of logistical and scientific requirements should not overshadow a discussion of ethical issues [4, 9, 10]. These points of view must be made explicit since failing to address ethical issues has serious consequences, including diminished public trust, low hospital staff morale, and ambiguity over duties and responsibilities [9, 11, 12].

The traditional pillars of medical ethics (such as beneficence, non-maleficence, justice, and autonomy) may not provide adequate guidance in extraordinary circumstances such as a pandemic. They must be complemented by different criteria to guide the allocation of scarce resources in a crisis. Currently, there are different polymorphs of ethical dilemmas, which need critical judgments, especially in the case of limited resources. For example, one study recommends and promotes resource allocation based on four additional ethical values: These include giving priority to the worst off, maximizing benefits yielded by scarce resources, treating people equally, and promoting and rewarding instrumental value [7]. Therefore, ethical values and their presentations need a critical analysis for acceptable and justifiable service delivery methods.

New ethical challenges continue to emerge as the pandemic continues to progress [13, 14]. For instance, COVID-19 has created ethical questions about the management of non-COVID-19 patients, especially patients who presented with complaints of upper respiratory infections (URTI), emergency cases, and dental problems [15, 16]. Patients with URTI show similar signs and symptoms to COVID-19 patients. These may create a problem in getting necessary support and treatment from health care settings. In addition, they will encounter stigma from the community, even from some health workers. At the same time, patients with dental problems admitted to the emergency department will not get adequate consultation and treatment, especially in settings where necessary PPEs are scarce.

In addition to the increased demand for PPE, there was an increased allegation of off-label prescribing and stockpiling of medicines such as some antibiotics, paracetamol, and hydroxychloroquine [17, 18]. This practice raised ethical issues regarding whether the pharmacist to dispense or not. In countries where most medicines are available without a prescription, the scenario will generate moral distress among health workers [19]. Therefore, the study aimed to explore the ethical dilemmas, and challenges in the management of patients with high-risk clinical services such as upper respiratory tract infections, dental complaints, and admission to the emergency department. Additionally, it also assessed the ethical challenges and its polymorphism in the allocation of health commodities that are directly used for the prevention and treatment of COVID-19.

## Methods

### Study setting and study design

A nationwide cross-sectional survey was conducted at randomly selected public healthcare facilities in Ethiopia. The study employed mixed methods (quantitative and qualitative) approach for data collection. The study adopted a qualitative case study design, which is used as an empirical inquiry that investigates a contemporary phenomenon in depth and within its real-life context to know how COVID-19 affected the ethical delivery of selected clinical services and allocation of health commodities directly used for prevention and treatment of COVID-19. This type of study approach allows for explaining, describing, or exploring in-depth, multi-faceted complex issues of ethical dilemmas in real-life settings.

### Study site selection and participant recruitment

Purposively selected healthcare facilities, COVID-19 treatment centers, quarantine, and COVID-19 testing centers found in Ethiopia were included. From the selected study site frontline health workers, coordinators, and supply chain managers were prospectively approached by the operational project lead to participate in the study. There were steps in which we tried to approach the prospective participants. The first step was a purposive selection of institutions of health facilities. In the second step, lists of professionals working in the selected study area and/or center were identified. From those identified health workers /coordinators, randomly selected study participants were approached to provide informed consent for participation. In areas where not possible to conduct face-to-face data collection, the data collection process took place through “online google forms’’ and the participant's email addresses. Data collectors and interviewers collected pertinent information from each study participant accordingly.

### Study population and period

In selected healthcare organizations, the target study group was health workers involved in treating COVID-19 patients, working in the dental clinic, emergency department, intensive care units, and Eye-Ear-Nose and Throat (EENT) clinic. Supply chain managers, COVID-19 emergency operation centers (EOC) coordinators, and other selected individuals, who were directly involved in the allocation of health commodities used for the prevention and treatment of COVID-19 were included. These individuals were selected from COVID-19 EOC, COVID-19 treatment centers, quarantine centers, pharmacies, emergency units, intensive care units, EENT clinics, and dental clinics. The study was carried out from March 06, 2021, to April 15, 2021.

### Data collection procedures

The participants were approached to explore their lived experiences, challenges, and ethical dilemmas that they encountered during the COVID-19 outbreak for their specific tasks. An in-depth interview with flexible probing techniques was used to collect data on the challenges encountered during service provision and health commodities distribution and use. A semi-structured interview guide that included open questions about the understanding of clinical ethics, the ethical issues participants encounter in clinical practice, the practical relevance of these issues, and how they deal with them during COVID-19 was used. Relevant details of each study participant were also collected. To maintain the participant's privacy, the interview took place in the office or a separate room in the health facility/institution. For the risk of COVID-19 infection that might happen during the survey and data collection. We followed the current infection prevention protocols recommended by the WHO & Ethiopian ministry of health and took all necessary protective measures (that includes the provision of personal protective equipment, social distancing, and related measures) for the study participants and as well for data collectors in the context of safeguarding and managing the potential risks.

### Data management and statistical analysis

Data were coded, entered using EPI data version 3.1, and analyzed using Stata version 15 statistical software. Responses were analyzed using descriptive statistics. Responses to the open-ended question were analyzed using a template analysis approach. The thematic approach and SWOT analysis were conducted to further assess the ethical issue, its main causes, and implications on the service delivery and containment of the pandemic. Key themes relating to each study participant’s ethical challenges and responses were identified.

### Ethical considerations and informed consent

Ethical clearance & approval was obtained from the institutional review board (IRB) of the Institute of Health of Jimma University (Reference number: JHRPG/1077/2021). The health facility director or head of the study center was informed about the purpose of the study to get agreement and cooperation before the start of the study. Written informed consent was taken from each participant after a clear orientation of the study objective. Strict confidentiality was assured through the anonymous recording and coding of questionnaires placed in a locked cabinet.

## Results

### Sociodemographic characteristics of study participants

Of the 285 frontline health workers to whom questionnaires were distributed, 217 responded (response rate 76.1%). Most respondents were male (53.5%) and young (mean age was 32, median age 25 years), and had less than 5 years of professional experience (ranging from 1 to 19 years). Regarding the respondents’ educational status, more than half of them had a first degree, while approximately one-third of them were specialists. Many had long working hours (mean ± SD; 63 ± 25 h in their current institution) and saw many patients during a week (Table 1).

**Table 1:** Sociodemographic characteristics of study participants and general information about the study setting

S. No	Variable	Category	Frequency (Percentage)
1.	Gender	Female	101(46.5)
		Male	116(53.5)
2.	Age of participants (years)	Mean ± SD	32 ± 21
		18–30	79(36.4)
		31–45	121(55.8)
		> 45	17(7.8)
3.	Educational level	Diploma	37(17.1)
		First degree	113(52.1)
		Masters (specialty) degree and above	67(30.8)
4.	Work experience(years)	< 2	57(26.3)
		3–5	92(42.4)
		> 5	68(31.3)
5.	Average work hours/week	Mean ± SD	63 ± 25
		≤ 40 h	29(13.4)
		40–100 h	121(55.8)
		> 100 h	67(30.8)
6.	Work position/area	Intensive care unit	47(21.7)
		Emergency department	63(29)
		Inpatient wards	67(30.9)
		Patient triage unit	27(12.4)
		Health commodity supply chain	13(6)
7.	Professional status (*n* = 217)	Nurse	96(44.2)
		Physician(medical doctor)	89(41)
		Dentist	17 (7.8)
		Pharmacist	8(3.7)
		Others^a^	7(3.3)
8.	The average number of patients /week (mean ± SD)		157 ± 85(50–850)
9.	Facility type(*n* = 19)	COVID-19 field hospital	6 (31.6)
		General Hospital	9(47.4)
		Tertiary hospital	10(52.6)

^a^Public health professionals, anesthesiologists

### Results from the quantitative analysis

#### Frontline health workers' experience in managing difficult medical decision-making during COVID-19

The twenty-four (24) ethically challenging situations were sorted according to how often the health workers experienced managing difficult medical decision-making during the COVID-19 report. These were grouped into eight major ethical themes.

Eleven of the specified challenging situations were experienced very frequently or frequently by more than half of the frontline health workers amid the COVID-19 pandemic. Respondents frequently reported encountering rationing dilemmas on health commodities directly used for the prevention and treatment of COVID-19. The most frequently encountered challenges concerned the allocation of those commodities; 83.9% agreed that they encountered ethical challenges very frequently or frequently to make difficult choices due to resource limitations. Almost all claimed that the limitation of resources directly used for treatment and prevention of COVID-19 such as PPE, ventilators, and ICU beds required them to make difficult choices 215( 99.1%), and 83.4% very frequently or frequently encountered dilemmas because patients were unable to pay for the preferred course of treatment.

Difficulties concerning doubts about helping or hurting the patient with the intervention, conflicting views in the family or concerns for effects on the family welfare, involuntary hospitalizations of suspected or confirmed COVID-19 patients, conflicting feelings on obligations to care for non-COVID-9 patients with dental and upper respiratory tract infection (URTI) complaints were all more frequently experienced. Most of the participants reported that most of their colleagues provided inappropriate care or unethical care because of a lack of inadequate knowledge in the prevention and treatment of COVID-19. From all major themes, ethical situations related to end-of-life issues were less frequently reported. Dilemmas concerning withholding or withdrawal of life-prolonging treatment of seriously ill or dying patients and requests for euthanasia or assisted suicide were reported to occur commonly among less than 15% of health workers (Table 2).

**Table 2:** Percentages of health workers, who experienced ethical challenges in managing difficult medical decisions

Situations	Very frequently	Frequently	Sometimes	Rarely	Never/Not applicable
A. **DOING GOOD OR HARMING**					
1. You worried if you were helping or hurting the patient with the interventions	50(23)	62(28.6)	51(23.5)	42(19.4)	12(5.5)
2. You felt that the patient's need for treatment was not in agreement with the patient's family needs or welfare	37(17)	46(21.2)	28(12.9)	92(42.4)	14(6.5)
3. You felt conflicted between your obligations to care for non- COVID-19 patients who have dental complaints	15(6.9)	41(18.9)	46(21.2)	81(37.3)	34(15.7)
4. You felt conflicted between your obligations to care for non- COVID-19 patients who have respiratory tract infection complaints	90(41.5)	59(27.2)	46(21.2)	13(6)	9(4.1)
5. You encountered involuntary hospitalization of suspected or confirmed COVID-19 patients	80(36.9)	50(23)	44(20.3)	24(11.1)	19(8.7)
B. **END-OF-LIFE ISSUES**					
1. You were asked to help a patient to have a voluntary euthanasia	0	0	6(2.8)	13(6)	198(91.2)
2. You cared for a terminally ill patient and the question of when to stop treatment or a "Do not resuscitate" order came up	25(11.5)	59(27.2)	18(8.3)	46(21.2)	69(31.8)
3. You were withholding (not starting) potentially a life-prolonging treatment for a seriously sick patient to prevent prolonged death and suffering	5(2.3)	7(3.2)	11(5.1)	13(6)	181(83.4)
4. You were withdrawing (removing) potentially life-prolonging treatment to a seriously sick patient to prevent prolonged death	9(4.1)	11(5.1)	15(6.9)	37(17.1)	145(66.8)
C. **ALLOCATION OF COVID-19 RESOURCES & HEALTH COMMODITIES**					
1. You felt you were over-treating patients, i.e. providing treatment or diagnostic tests they could not benefit from	8(3.7)	15(6.9)	63(29)	72(33.2)	59(27.2)
2. You were restricting treatment to a patient to give those resources to someone who could benefit more (i.e. hospital/ICU bed, ventilator, medication)	37(17.1)	98(45.2)	52(23.9)	23(10.6)	7(3.2)
3. The preferred course of treatment was not pursued because of a patient's inability to pay	108(49.8)	73(33.6)	20 (9.2)	13(6)	3(1.4)
4. The limitation of resources used for the treatment and prevention of COVID-19 required you to make a difficult choice	113(52.1)	69(31.8)	28(12.9)	5(2.30)	2(0.92)
5. There was significant disagreement among health workers on continuing the treatment of the patient due to a lack of resources	50(23)	93(42.9)	35(16.1)	29(13.4)	10(4.6)
6. Been so troubled by limited resources that you regretted your choice of profession	72(33.2)	89(41)	27(12.4)	16(7.4)	13(6)
7. Seen a situation where a patient was infected with COVID-19 as a result of limited resources in the health care system	11(5.1)	37(17)	29(13.4)	87(40.1)	53(24.4)
D. **CONFLICTING INTERESTS**					
1. Your preferred course of treatment conflicted with institutional policies, professional codes of ethics, or laws	14(6.5)	28(12.9)	33(15.2)	89(41)	53(24.4)
E. **DISAGREEMENT WITH PATIENT OR FAMILY**					
1. There was significant disagreement among family members on the continuing treatment of the patient	6(2.7)	16(7.4)	47(21.7)	72(33.2)	76(35)
2. A patient's cultural or religious views conflicted with your proposed course of treatment	18(8.3)	20(9.2)	51(23.5)	61(28.1)	67(30.9)
F. **PATIENT CAPACITY TO CONSENT**					
1. You cared for patients that were not in a state to decide for themselves (like unconscious/ disabled), and you had to decide for them	25(11.5)	58(26.7)	71(32.7)	42(19.4)	21(9.7)
G. **UNETHICAL OR INAPPROPRIATE CARE**					
1. You witnessed that a colleague was not acting according to professional standards(like not being honest, fair, responsible, and respectful)	33(15.2)	49(22.6)	81(37.3)	35(16.1)	19(8.8)
2. You came across colleagues that compromised the quality of care for the fear of COVID-19	38(17.5)	72(33.2)	57(26.3)	37(17)	13(6)
3. You came across colleagues not providing appropriate care because of inadequate medical knowledge and skills in the prevention and treatment of COVID-19	44(20.3)	57(26.3)	74(34.1)	35(16.1)	7(3.2)
H. **DISCLOSURE OR CONFIDENTIALITY ISSUES**					
1. You were in doubt if a diagnosis of COVID-19 should be disclosed to the patient	64(29.5)	33(15.2)	77(35.5)	25(11.5)	18(8.3)

*COVID-19* Coronavirus Disease-2019

#### Difficulty in the provision of essential clinical service delivery

**Fig. 1:**
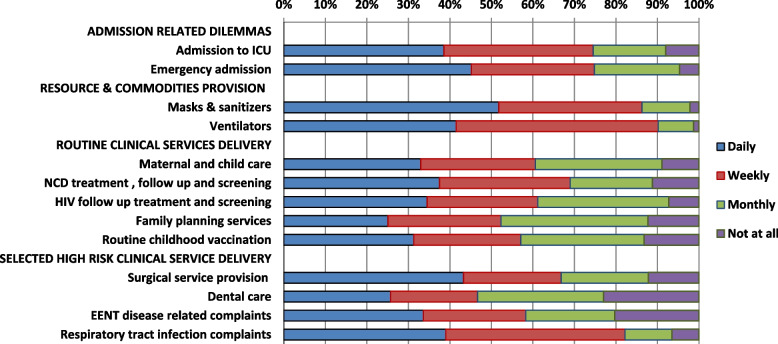
Health care professionals' difficulty in the provision of essential clinical services during COVID-19. NCD: Non-communicable disease; ICU: intensive care unit; EENT: Eye, Ear, Nose, throat; HIV: Human immunodeficiency virus

Frontline health workers were asked a series of questions containing the following stem: "During the COVID-19 pandemic, how often (daily, weekly, monthly, and not at all) were you unable to obtain/provide the following services for your patients when you thought they were necessary?" The frequency of difficulty in the provision of essential clinical services varied between 77% and 98.7% for different services. Difficulty in the provision of intensive care unit and emergency admission, availability of ventilators, masks, and sanitizers for service delivery, and treatment of patients with complaints of respiratory tract infections were most frequently experienced on a daily or weekly basis. More than half of the study participants reported that they had encountered difficulty in the provision of access to family planning services, maternal & child care, childhood immunization, and chronic disease follow-up and screening encountered on a daily or weekly basis (Fig. 1).

#### Strategies for rationing health commodities and admission to health facilities

**Fig. 2:**
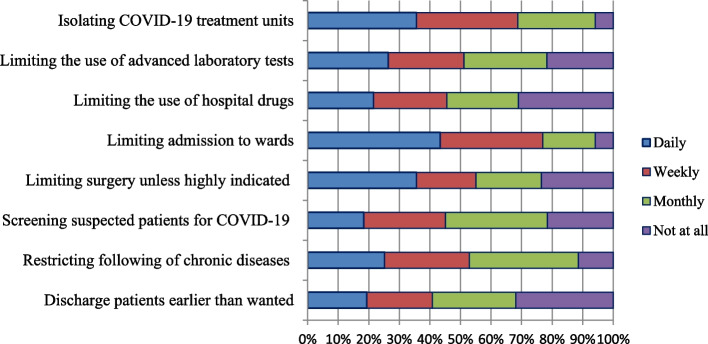
Strategies used by a health care professionals/managers to ration the health commodities and admission to health facilities

Frontline health workers use different rationing strategies for health commodities, admission, and delivery of clinical services. To identify different strategies of rationing, we asked participants a series of questions containing the following stem: "During the COVID-19 pandemic, how often (daily, weekly, monthly, not at all) did you try to save health care costs for your health care facility by …”?. Isolating COVID-19 treatment units and limiting admission were the most frequent rationing strategies used by two-thirds of health workers on a daily or weekly basis. Discharging patients earlier than wanted, limiting the use of hospital drugs, and screening suspected patients for COVID-19 were less often rationed by frontline health workers. More than half of the participants used limiting chronic disease follow-up and surgical procedures on a routine basis as one of the rationing strategies to prevent transmission of COVID-19 and the burden on healthcare facilities (Fig. 2).

### Results from the qualitative analysis

Of the 217 respondents, 51 responded to the open-ended question *“If you have experienced any of the ethical situations, or any other striking ethical dilemma, can you please briefly describe some of the ethical challenges you are facing/have faced during the COVID-19 outbreak? (Ethical challenges are situations that give you cause for professional concern, or when it is difficult to decide what is the right action to take. This may be a situation facing you, or something you have come to hear about from others).”*Most of them provided one or several examples described in detail, while others presented bullet-point lists of dilemmas they had experienced themselves or situations they found ethically challenging, in general. Examples of all the categories of dilemmas we had included in the survey from the rationing of health commodities and delivery of clinical services during the COVID-19 pandemic were presented.

#### Rationing dilemmas on health commodities during the COVID-19 pandemic

As in the quantitative question, the most frequent examples were ethical challenging situations concerning the allocation of resources, especially those used for the prevention and treatment of COVID-19. Several health workers mentioned that they encountered rationing dilemmas on health commodities directly used for the prevention and treatment of COVID-19. Most frontline health workers described “*limitation of personal protective equipment is the major challenge*”. No examples concerned with ethical situations are mentioned under the end-of-life issues. The problem is about situations in which patients had competing needs in the face of insufficient admission beds. Even though the distributive dilemma is also common during normal situations, health workers encountered these ethical challenges more frequently during an emergency COVID-19 pandemic. One participant described how this is a daily part of his job in ICU admission.

*“………….It is my daily duty hours experience to deny patients (emergency patients) resources of care, especially ICU admission beds, to prioritize one over another based on the hemodynamic situation of the patients. Since resources are never enough to accommodate every patient”.*

Some respondents explained how the strategy of “first-come, first-served” was typically used and how they disagreed with it.

*“….……we should do everything possible to minimize the damage of the COVID-19 pandemic, but it’s also time to decide how to best allocate scarce medical resources, which is common in our settings. It is difficult to give priorities to those who benefit more, as several patients had already registered before their admission”.*

However, one of the ICU physicians raised the ethical challenges from the old ''first-come, first-served'' principle, which is a common method in the allocation of medical resources.

*"………..still, we can use the principle with its minimal drawbacks when health system capacity is sufficient to provide adequate care to all comers in due time. However, in case of an increased surge of COVID-19 cases, it will not work to treat everyone equally. I fear some of the individuals who know (relatives) or approach the health care professional working in the facility may get the service easily”.*

Some of the participants reported a lack of clear ethics guidelines for the provision of some of the clinical services. They think the current national clinical management guidelines for COVID-19 will not address the ethical challenges encountered adequately during the crisis. One of the gynecologists described associated ethical challenges as:

*“………There was no clear guideline that will support the clinical decision if major ethical dilemmas arise during these uncertain times. For example, we have no surgical triage guidelines to evaluate the patient for surgical interventions. Mostly we depend on the individual experience or a team of experts’ decision to perform surgical procedures for the patients”. What I remember is the surgical intervention for termination of pregnancy (abortion), which is not emergent but if postponed pushes termination time to a later gestational age, which increases procedural risks to patients".*

#### Dilemmas concerning the delivery of essential clinical services

Similar to the report in the quantitative section, most of the health workers reported that they encountered difficulty in the provision of maternal and child care, NCD and HIV follow-up treatment and screening, routine childhood vaccination, and family planning services during COVID-19.

Based on Klein’s [20] six (6) forms of rationing strategies, frontline health workers used different strategies. One of the rationing methods would be selection or service termination. Those health workers working in patient triage, emergency, and ICU admission have been selecting those patients who were most likely to benefit from hospital admission or raise the threshold of eligibility for admission. Similarly, nearly three-fourths of participants limit admission as one of the rationing strategies on the quantitative data. As one described:

“………*we usually tried to save available emergency & ICU beds based on the patient's conditions and available unoccupied beds. The availability of PPE, sanitizers, and beds, as the patients’ medical-related considerations including the patient’s comorbidity, especially patients with chronic respiratory disorders, such as asthma, chronic obstructive lung disease, and patients with diabetes mellitus sometimes will be considered as admission criteria*.”

The fear of COVID-19 transmission affected the care of patients with chief complaints of respiratory symptoms. As depicted in quantitative data, nearly one-third of health workers encountered dilemmas in the provision of clinical care for patients with such complaints. One of the emergency nurses working in the triage area explained:

*"…..Especially in the first phase of COVID-19 almost all of us were not happy to evaluate patients with fever, cough, and patients with symptoms of acute febrile illness. Their symptoms are similar to what we see or hear about in patients with COVID-19. Even such kind patients experienced great anxiety and have been not getting the proper care for their complaints. "*

## Discussion

COVID-19, which began in China in December 2019, has spread rapidly around the world, infecting over 137 million people and killing approximately 3 million people as of mid-April 2020 [21, 22]. Competence is a central ethical requirement for routine clinical practice [23]. In general, experts should not perform functions beyond the limits of expertise. Any actual, potential, or apparent competing loyalty must be disclosed to the patient [24–26]. Public health emergencies have an impact on each of these ethical standards. In a pandemic, the standard rules of healthcare services no longer apply [27–29]. Health workers are facing existential decisions: Who will they treat, and who will they let die? Healthcare facilities' capacity is overwhelmed and medical staff is falling ill from the virus. They simply can't treat everyone. During severe pandemics, it may be necessary to call upon health professionals and even non-health professionals to temporarily and occasionally perform tasks that lie outside the bounds of their certification (or even competence).

During COVID-19 there were three major identified ethical duties of healthcare leaders responding to COVID-19; duty to plan (managing uncertainty), duty to safeguard(supporting workers and protecting vulnerable populations), and duty to guide(contingency levels of care and crisis standards of care) [30]. The current study showed that in all the surveyed healthcare facilities frontline line health workers experienced ethical difficulties. There was a less frequent occurrence of patient disagreement on the proposed course of treatment for non-religious or cultural reasons, disagreement among family members on the continuing treatment of the patient, and more frequent doubt not disclosing the diagnosis of COVID-19 among Ethiopian frontline health workers. Such kinds of ethical situations occurred at different healthcare delivery sites. For example, a previous study from four European countries (Norway, Switzerland, Italy, and the UK [31] and Ethiopia [32] showed similar reports. This is an implication for a more paternalistic model of healthcare delivery, in which the health workers are entrusted with decisions.

In the current study, nearly three-quarters of health workers were so troubled by limited resources that they regretted their choice of profession. Similar studies from Israel [33, 34], Jordan [35], the USA [36], and Italy [37, 38] showed that the scarcity of health commodities directly used for the prevention and treatment of COVID-19 scared health workers to care for COVID-19 patients. The shortage of healthcare resources, as well as health workers, is well-documented in low-income countries, including Ethiopia before COVID-19, due to various causes such as scarcity of supplies, poor healthcare infrastructure, limited ICU facilities, and lack of access to guidelines and protocols.

The scarcity of healthcare resources particularly in developing countries may create ethical dilemmas. This may include the need to provide care and treatment for more severely ill patients while delaying treatment for others who are in a better condition [39]. The need to take such decisions may cause some health workers to experience moral injury or mental health problems [40]. Almost all of the study participants reported that the limitation of resources used for the treatment and prevention of COVID-19 required them to make a difficult choice. The main concern of the COVID-19 pandemic is that the disease burden may exceed the healthcare resources that are available for treating patients. Even in developed countries, there was a concern that healthcare systems would be overwhelmed if COVID-19 cases increase dramatically [41]. For example, in the USA, Italy, and South Korea, there were not enough N95 masks, ventilators, and ICU beds a shortage in hospitals, which leads to many deaths [42–46]

About half of the participants reported that they encountered health workers not providing appropriate care because of inadequate medical knowledge and skills in the prevention and treatment of COVID-19. This might be related to the scarcity of healthcare resources, training, and a low number of health workers. Healthcare systems in developing countries face major problems during this time and are unlikely to offer the care needed [45]. Indeed, the requirements for ethical justification related to emergency preparedness are very stringent, the necessary changes are often destructive and consequential, and can be economically costly. Public health emergency creates great stress, especially for doctors who are not used to working in emergency conditions with limited resources. A sound ethical framework for healthcare in public health emergencies must balance the obligation of patient-centered care – with a focus on clinical ethics under normal circumstances [47, 48].

Clinical care is patient-centered, with the ethical course of action aligned, as far as possible, with the preferences and values of the individual patient. A public health emergency, such as a surge of persons seeking health care as well as critically ill patients with COVID-19 or another severe respiratory illness, disrupts normal processes for supporting ethically sound patient care. In the current study, more than three fourth of front-line health workers encountered difficulty in the provision of delivery essential clinical services for different services. Difficulty in the provision of intensive care unit and emergency admission, availability of ventilators, masks, and sanitizers for service delivery, and treatment of patients with complaints of respiratory tract infections were most frequently experienced on a daily or weekly basis. This is similar to studies from Hong Kong [49], India [50], Italy [38], and the USA [51]. Similarly, more than half of the frontliners reported that they had encountered difficulty in the provision of access to family planning services, maternal & child care, childhood immunization, and chronic disease follow-up and screening encountered on a daily or weekly basis. This is in line with multiple studies across the world such as in the USA [52], Qatar [53], India [52], and the UK [54].

COVID-19 created challenges specific to women's health and highlights the potential devaluation of women's health with the resulting long-term consequences. In the current study, more than 50% of study participants reported that there encountered a problem in the delivery of family planning services and maternal & child care. These showed ethical challenges for women's healthcare during the COVID-19 pandemic. In line with a report from the USA [55], disproportionately disadvantaged women in the delivery of essential services. Despite the scarce data available on maternal or fetal morbidity [56, 57], most of the maternal and child health clinic (MCH) services were postponed, due to fear of pandemic transmission.

Similar to a report from the USA [55], COVID-19 posed difficulty in the provision of surgical services. Nearly two-thirds of frontline health workers reported that they were unable to provide routine surgical services for their clients. As per international recommendations deferral of elective, or non-urgent, surgeries to limit infectious exposure and conserve medical equipment, especially personal protective equipment (PPE), in settings with a high burden of COVID-19 [58–60].

As the coronavirus disease 2019 (COVID-19) pandemic intensifies, shortages of ventilators have occurred across the world, especially in countries with the highest burden of COVID-19 cases such as Italy and the USA [7, 61]. Likewise, in our study, more than 90% of front-line health workers encountered a dilemma to access ventilators for needy patients. This poses difficulty in ICU admission and patient management. Health needs created by the coronavirus pandemic go well beyond the capacity of the hospital found in developed countries [62]. This definitely will cause a significant ethical dilemma in the rationing of essential commodities such as mechanical ventilators, PPE, and others in resource constraint countries, such as Ethiopia due to their background of weak health system financing and development. Public health practice aims to promote the health of the population by minimizing morbidity and mortality through the prudent use of resources and strategies. Limiting personal rights and preferences may be required to ensure the health of the population, especially in emergencies. In the event of a disaster, health workers can face difficult questions about who has limited life-saving resources. Public health ethics guide us in balancing this tension between individual and group needs. The ethical principles that should guide decision-making have been considered by expert panels but have not been well explored with the front-line health workers at health facilities.

### Strength and limitation of the study

The present study has several strengths. To the best of the authors' knowledge, this is one of the first studies to evaluate the ethical challenges in clinical service delivery and health commodity allocation that are directly used for COVID-19 prevention and treatment. It employed the mixed methods approach, which will support the quantitative data about the ethical issues and explored the lived experience of front-line health workers. In addition, it is a nationwide survey, which will provide a national data on the topic. However, there may be some possible limitations in this study. Primary care health facilities (i.e., primary hospitals and health centers) were not included in the study. These facilities have significant resource availability and service delivery problem before COVID-19, and which will be fueled during the pandemic. Additionally, the study did not consider the perspectives of patients and their families, who may have their own ethical concerns and considerations regarding healthcare delivery during the pandemic. Future research could explore these perspectives and provide a more comprehensive understanding of the ethical challenges faced in healthcare during COVID-19.

## Conclusions

Front-line health workers have encountered ethically challenging situations during COVID-19 pandemic. More than half of health workers reported ethical challenges when rationing resources and providing various clinical services such as family planning, maternal and childcare, immunization, and chronic care. A critical ethical issue in the COVID-19 pandemic is the equitable distribution of limited available resources among patients. With limited resources such as ventilators and hospital beds, healthcare providers have been faced with the difficult task of deciding who gets access to these resources and who doesn't. Outpatient and inpatient health care services, including routine hospitalization and elective surgery, have been severely curtailed or postponed. Overall, the COVID-19 pandemic has presented numerous ethical challenges for healthcare providers, highlighting the importance of ethical considerations in healthcare delivery. By being aware of these dilemmas and having policies in place to address them, healthcare providers can ensure that they are providing the best possible care to their patients while upholding ethical standards. Further exploration and refinement of policies are necessary to ensure that ethical considerations are fully integrated into the delivery of healthcare during the pandemic.

## Data Availability

Data are available from the corresponding author upon reasonable request.

## Abbreviations

COVID-19: 2019 Coronavirus Disease
EFDA: Ethiopian Food, and Drug Authority
ENT: Ear, Nose, and Throat
EOC: Emergency Operation Center
HIV: Human Immunodeficiency Virus
ICU: Intensive Care Unit
NCD: Non-Communicable Disease
PPE: Personal Protective Equipment
URTI: Upper Respiratory Infections
